# Distinct serum steroid profiles between adrenal Cushing syndrome and Cushing disease

**DOI:** 10.3389/fendo.2023.1158573

**Published:** 2023-05-16

**Authors:** Chang Gao, Li Ding, Xiaona Zhang, Menghua Yuan, Shaofang Tang, Wei Li, Yuanyuan Ye, Ming Liu, Qing He

**Affiliations:** ^1^ Department of Endocrinology and Metabolism, Tianjin Medical University General Hospital, Tianjin, China; ^2^ Department of Endocrinology, Wuhan No.1 Hospital, Tongji Medical College, Huazhong University of Science and Technology, Wuhan, China

**Keywords:** dehydroepiandrosterone sulfate (DHEA-S), serum steroids, mass spectrometry, orthogonal partial least squares discriminant analysis (OPLS-DA), Cushing syndrome

## Abstract

**Background:**

Differentiating between adrenal Cushing syndrome (adrenal CS) and Cushing disease (CD) can be challenging if there are equivocal or falsely elevated adrenocorticotropic hormone (ACTH) values. We aim to investigate the diagnostic value of serum steroid profiles in differentiating adrenal CS from CD.

**Method:**

A total of 11 serum steroids in adrenal CS (*n* = 13) and CD (*n* = 15) were analyzed by liquid chromatography with tandem mass spectrometry (LC-MS/MS). Age- and gender-specific steroid ratios were generated by dividing the actual steroid concentration by the upper limit of the relevant reference range. A principal component analysis (PCA) and an orthogonal partial least squares discriminant analysis (OPLS-DA) were performed.

**Results:**

The PCA and OPLS-DA analyses showed distinct serum steroid profiles between adrenal CS and CD. Dehydroepiandrosterone sulfate (DHEA-S), dehydroepiandrosterone (DHEA), and androstenedione ratios were identified as biomarkers for discrimination by variable importance in projection (VIP) in combination with *t*-tests. The sensitivity and specificity of DHEA-S ratios <0.40 were 92.31% (95% CI 64.0%–99.8%) and 93.33% (95% CI 68.1%–99.8%), respectively, in identifying adrenal CS. The sensitivity and specificity of DHEA ratios <0.18 were 100% (95% CI 75.3%–100.0%) and 100% (95% CI 78.2%–100.0%), respectively, in identifying adrenal CS.

**Conclusion:**

Our data support the clinical use of the DHEA-S and DHEA ratios in the differential diagnosis of adrenal CS and CD, especially when falsely elevated ACTH is suspected.

## 1 Introduction

Cushing syndrome (CS) is a disease characterized by endogenous hypercortisolism. It can be adrenocorticotropic hormone (ACTH) dependent when it results from excessive ACTH production by a pituitary corticotroph adenoma (Cushing disease, CD), or by an extrapituitary tumor secreting ACTH (ectopic ACTH syndrome) or CRH (ectopic CRH syndrome). CS can also be ACTH-independent when it results from cortisol overproduction by adrenocortical tumors (adrenal CS). As surgery is the primary treatment, after a diagnosis of CS is confirmed, further diagnostic tests to determine the exact etiology should be performed.

Plasma ACTH is the first diagnostic test in differentiating between ACTH-dependent CS and ACTH-independent CS (1, 2). Lower levels of plasma ACTH are indicative of ACTH-independent CS, and an ACTH level of < 2.2 pmol/L (10 pg/mL) can usually exclude the diagnosis of ACTH-dependent CS. However, previous studies have reported ACTH values that are not fully suppressed in adrenal CS patients (3, 4), leading to difficulties in differentiating between ACTH-dependent CS and ACTH-independent CS. Moreover, spurious elevated ACTH results due to heterophile antibody interference led to misdiagnosis or unnecessary testing (5, 6). Owing to the impact of plasma ACTH values on clinical practice, the results must be interpreted with caution (4).

The adrenal gland produces multiple steroids and the latest research suggests distinct steroid profiles between CS subtypes. Using simultaneous liquid chromatography and tandem mass spectrometry (LC-MS/MS) measurements of 15 adrenal steroids in plasma, Graeme Eisenhofer et al. generated a 10-steroid panel that could provide optimal discrimination of three subtypes of CS (7). We analyzed serum steroids in CS patients based on CD or adrenal CS grouping, aiming to unearth steroids that contributed to the differentiation of adrenal CS from CD and evaluate their diagnostic utility in differentiating adrenal CS from CD.

## 2 Materials and methods

### 2.1 Subjects

**Figure 1:**
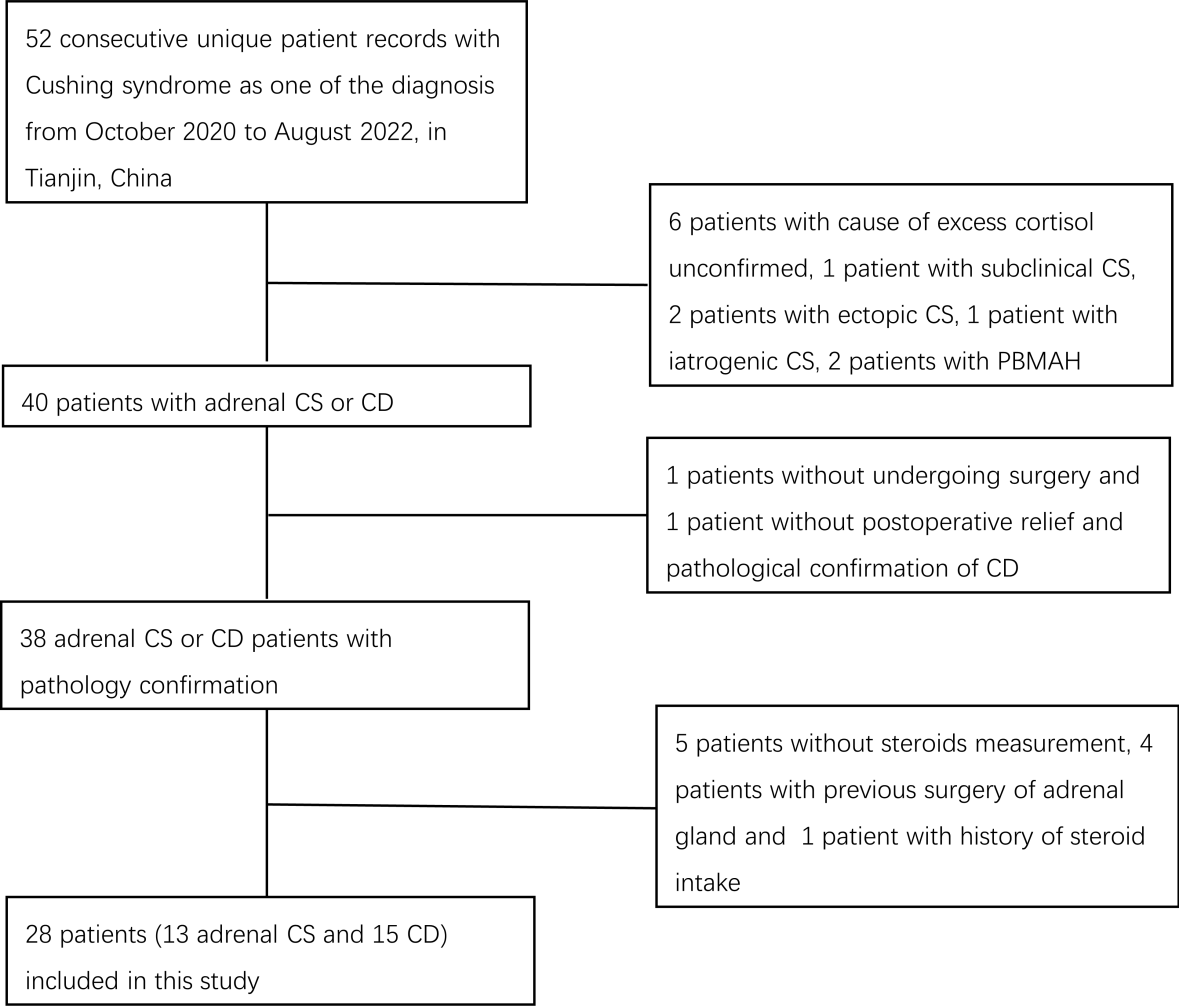
Flow chart of identification of study population.

We retrieved records of patients diagnosed consecutively with Cushing syndrome between October 2020 and August 2022 at the Department of Endocrinology and Metabolism at Tianjin Medical University General Hospital. The diagnosis and differential diagnosis between Cushing syndrome subtypes were made by hormone evaluations such as 24-h urinary free cortisol (UFC), ACTH, low-dose dexamethasone suppression test (LDDST), high-dose dexamethasone suppression test (HDDST), desmopressin (DDAVP) stimulation test, and bilateral inferior petrosal sinus sampling (BIPSS) measurements, and radiological imaging findings in accordance with current guidelines (8). Patients with excess cortisol with an unconfirmed cause and patients with subclinical CS, ectopic CS, iatrogenic CS, or CS due to bilateral macronodular adrenal hyperplasia were not included. We did not include the two ectopic CS patients because one patient had undergone adrenal gland surgery before steroid measurement and one patient did not achieve total tumor resection and clinical remission and was therefore on medical therapy. The diagnosis of adrenal CS or CD was confirmed by postsurgical pathological examination. One patient who did not undergo surgery and one patient without postoperative relief and pathological confirmation of CD were excluded. Patients with a history of steroid intake in the previous 5 years and patients lacking steroid measurement results were also excluded. Finally, 28 patients, including 15 patients with CD and 13 patients with adrenal CS, were enrolled (**Figure 1**). This study was approved by the Institutional Review Board of Tianjin Medical University General Hospital with a waiver of individual patient consent.

### 2.2 Methods

To evaluate the clinical usefulness of the differential diagnoses of adrenal CS and CD, we estimated the following serum steroid hormones: progesterone, 17-OH-progesterone, dehydroepiandrosterone (DHEA), DHEA-S, androstenedione (A4), testosterone, 11-deoxycorticosterone (DOC), 11-deoxycortisol (11-DOC), cortisol, corticosterone, and cortisone. Blood samples for steroid measurement were taken in the morning after at least 8 h of fasting. A steroid testing kit (Hangzhou Calibra Diagnostics Co., Ltd., Zhejiang, China) was used for sample preparation. A volume of 100 µL of EHA extractant was added to 100 µL of serum. After vortexing for 10 min, the mixture was centrifuged at 14,000 g and 4°C for 10 min. A volume of 100 µL of supernatant was analyzed by a Jasper™ high-performance liquid chromatography system connected to an AB SCIEX Triple Quad™ 4500 MD mass spectrometer with a heated nebulizer ionization source in positive ion mode. The MultiQuant™ MD 3.0.3 software was used to quantify the data. Six calibration standards and two quality control samples were included for each set of samples. Using linear regression with 1/x^2^ weighting, the correlation coefficients of the peak intensities of analytes to the internal standards were all greater than 0.99.

Plasma ACTH, cortisol (besides the sample measured by LC-MS/MS), and 24-h urinary free cortisol were measured by chemiluminescent enzyme immunoassay (Siemens Healthcare Diagnostics Inc., Erlangen, Germany). Renin and aldosterone were measured by chemiluminescence immunoassay (DiaSorin, Saluggia, Italy).

### 2.3 Statistical analysis

Chi-squared tests were used for categorical variables and Mann–Whitney *U*-tests were used for quantitative variables. To rule out the influence of sex and age on steroid measurements, steroid data were divided by sex- and/or age-specific upper cutoff values established elsewhere (9) to generate steroid ratios. Steroid ratios were introduced into the SIMCA software (version 14.1; Umetrics, Sweden) for principal component analysis (PCA) and orthogonal partial least squares discriminant analysis (OPLS-DA) analysis. Data were logarithmically transformed, and Pareto scaling was conducted before model construction. *R* ^2^ and *Q* ^2^ were utilized to evaluate goodness of fit and the predictive ability of the constructed model, respectively. A permutation test with 200 permutations was conducted to evaluate the over-fitness of the model. S-plots, variable importance in the projection (VIP) scores, and *t*-tests were used to select potential biomarkers.

A receiver operating characteristic (ROC) curve analysis was performed to test the usefulness of DHEA-S, DHEA, androstenedione, and their ratios as markers for differentiating between adrenal CS and CD. The optimal cutoff values were determined using the Youden index (sensitivity + specificity − 1). The sensitivity and specificity for each marker were derived from the optimal cutoff value. Statistical analyses were performed using SPSS, version 25, and the MedCalc statistical software (version 20.022). A *p*-value <0.05 was considered statistically significant.

## 3 Results

### 3.1 Patient characteristics

No significant differences in sex and age were observed between the adrenal CS and CD groups (**Table 1**). Compared with the adrenal CS patients, those with CD exhibited higher basal serum cortisol (median, 37.40 µg/dL vs. 27.90 µg/dL; *p* = 0.005), basal plasma ACTH (median, 115.00 pg/mL vs. 5.00 pg/mL; *p* < 0.001), and 24-h UFC (median, 579.60 µg/24 h vs. 226.60 µg/24 h; *p* = 0.003). No significant differences in aldosterone and renin were observed. No significant differences in body mass index were observed, and patients with Cushing disease had nominally higher fasting blood glucose [5.00 (4.20–14.50) mmol/L vs. 4.50 (3.50–6.60) mmol/L; *p* = 0.060]. There was no significant difference in the percentages of patients with hypertension, diabetes mellitus, or smoking between the two groups.

**Table 1:** Baseline characteristics and biochemical test results of patients with adrenal Cushing syndrome and Cushing disease.

Characteristic	Adrenal Cushing syndrome (n = 13)	Cushing disease (n = 15)	p-value
Gender (F/M)	12/1	12/3	0.600
Age	36 (25–67)	36 (21–69)	0.683
Basal serum cortisol (µg/dL) (normal range 5.00–25.00 µg/dL)	27.90 (11.30–46.40)	37.40 (26.90–69.50)	0.005
Basal plasma ACTH 8:00 (pg/mL) (normal range 0.00–46.00 pg/mL)	5.00 (5.00–143.00)	115.00 (23.20–211.00)	< 0.001
24-h UFC (µg/24 h)	226.60 (56.24–597.50)	579.60 (111.60–1,550.00)	0.003
Serum cortisol after 1 mg of DST (µg/dL)	26.60 (11.70–40.20)	27.60 (3.35–50.00)	0.830
Serum cortisol after LDDST (µg/dL)	28.10 (16.70–38.80)	26.05 (2.13–59.90)	1.000
Serum cortisol after HDDST (µg/dL)	24.60 (15.70–29.70)	13.90 (1.38–50.00)	0.087
Aldosterone (ng/dL) (normal range 3.0–23.6 ng/dL)	4.30 (1.60–24.30)	4.00 (2.80–8.30)	0.314
Renin (µIU/mL) (normal range 2.8–39.9 µIU/mL)	5.10 (0.70–56.70)	4.85 (0.80–22.30)	0.512
Fasting glucose (mmol/L)	4.50 (3.50–6.60)	5.00 (4.20–14.50)	0.060
Body mass index (kg/m^2^)	24.15 (20.31–39.52)	24.44 (17.42–39.84)	0.728
Hypertension (%)	9 (69.2%)	10 (66.7%)	1.000
DM (%)	6 (46.2%) : 7 (53.8%)	7 (46.7%) : 8 (53.3%)	1.000
Smoking (%)	2 (15.4%)	3 (20%)	1.000

Data are expressed as a median (minimum–maximum) or number (%).

ACTH, adrenocorticotropic hormone; UFC, urinary free cortisol; LDDST: low-dose dexamethasone suppression test; HDDST, high-dose dexamethasone suppression test; DM, diabetes mellitus.

ACTH levels < 10 pg/mL, identified in 11 out of 13 patients with adrenal CS, corresponded to a sensitivity and specificity of 84.6% and 100%, respectively.

### 3.2 Steroid profiles and pattern recognition of adrenal Cushing syndrome and Cushing disease

Plasma concentrations of 11 steroids in patients with adrenal Cushing syndrome and Cushing disease are shown in **Table 2**. Serum steroid levels of testosterone (*p* = 0.037), dehydroepiandrosterone (*p* < 0.001), cortisol (*p* = 0.005), androstenedione (*p* < 0.001), dehydroepiandrosterone-sulfate (*p* < 0.001), and corticosterone (*p* = 0.019) were significantly lower in the adrenal CS group than in the CD group. There were no significant differences in serum levels of progesterone, 17-OH-progesterone, 11-deoxycorticosterone, 11-deoxycortisol, and cortisone between the two groups.

**Table 2:** Plasma concentrations of 11 steroids in patients with adrenal Cushing syndrome and Cushing disease.

Steroid	Adrenal Cushing syndrome	Cushing disease	p-value
T	152.02 (25.42–533.54)	218.50 (78.00–946.73)	0.037
DHEA	536.00 (111.25–907.47)	4,313.22 (1,252.00–8,610.71)	< 0.001
P	100.00 (8.00–5,858.72)	100.00 (8.00–262.47)	0.717
Cortisol	163,283.36 (91,838.13–243,000.00)	246,827.91 (86,980.52–398,047.00)	0.005
17-OHP	242.12 (72.41–1,644.37)	450.96 (138.61–1,080.92)	0.118
A4	437.46 (136.10–1,356.26)	1143.00 (489.00–3,050.45)	< 0.001
DOC	80.00 (35.00–276.45)	80.00 (15.22–127.52)	0.440
11-DOC	981.02 (386.51–3,543.56)	613.05 (179.89–1,859.94)	0.118
DHEA-S	0.30×10^6^ (0.07×10^6^–1.99×10^6^)	3.59×10^6^ (0.73×10^6^–7.05×10^6^)	< 0.001
Cortisone	16,701.13 (11,141.08–23,100.00)	21,268.13 (15,282.90–35,736.22)	0.118
Corticosterone	2,880.00 (885.66–5,951.02)	4,995.36 (1,060.00–13,226.76)	0.019

Data are expressed as medians and ranges in pg/mL.

T, testosterone; DHEA, dehydroepiandrosterone; P, progesterone; 17-OHP, 17-OH-progesterone, A4, androstenedione; DOC, 11-deoxycorticosterone; 11-DOC, 11-deoxycortisol; DHEA-S, dehydroepiandrosterone-sulphate.

**Figure 2:**
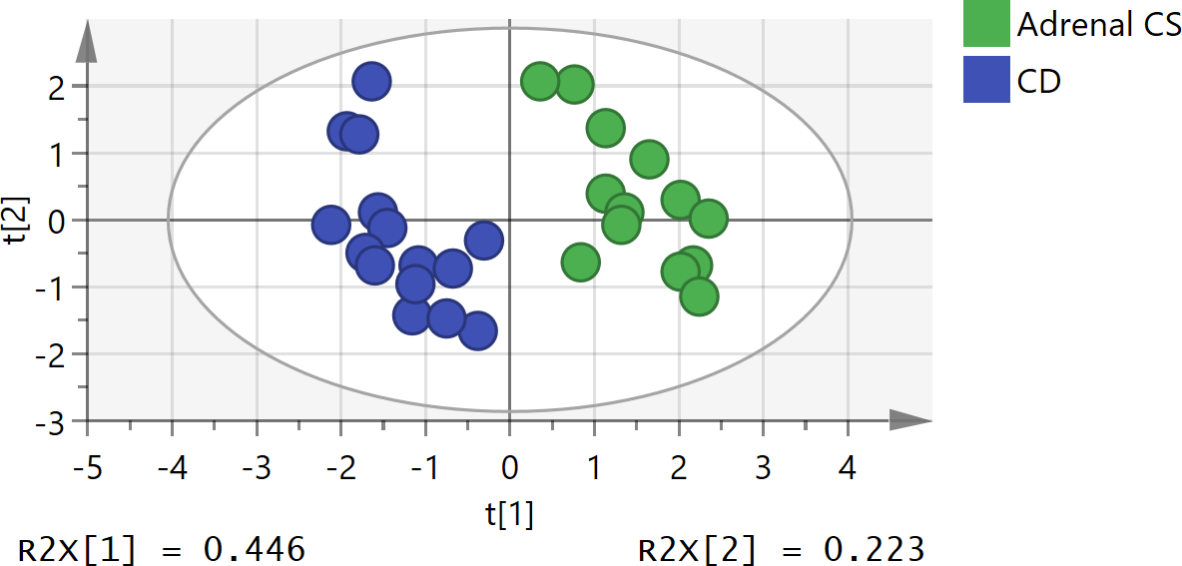
Principal component analysis (PCA) score plot of adrenal Cushing syndrome (adrenal CS) group and Cushing disease (CD) group. The *x-* and *y*-axes represent the first principal component and the second principal component after PCA, respectively. The model parameters were *R* ^2^ *X* = 0.79 and *Q* ^2^ = 0.36. PCA, principal component analysis.

The unsupervised PCA method was applied to sex- and age-adjusted basal serum steroids of the adrenal CS and CD patients. The score plot (*R* ^2^ *X *= 0.79 and *Q* ^2^ = 0.36; **Figure 2**) showed some separation of the adrenal CS patients and CD patients. The samples are dispersed, reflecting the heterogeneity of steroid profiles of CS patients. There were no strong outliers.

**Figure 3:**
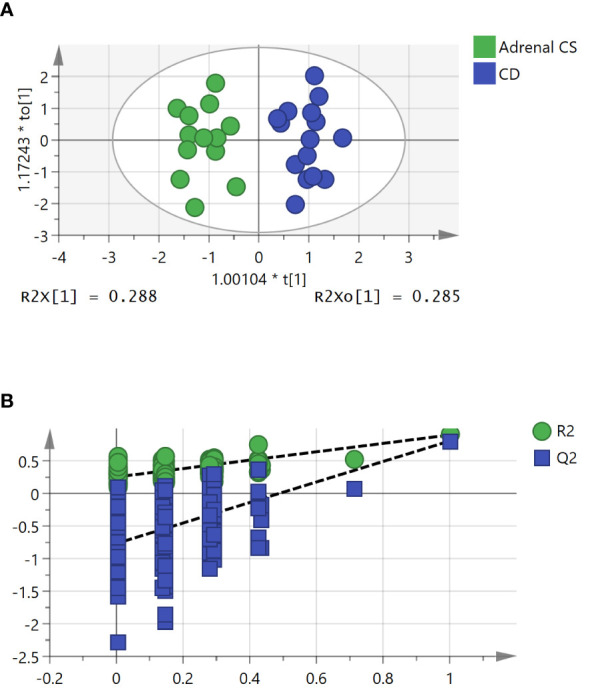
The orthogonal partial least squares discriminant analysis (OPLS-DA) model for the classification of adrenal Cushing syndrome (adrenal CS) and Cushing disease (CD). **(A)** Score plot of first predictive component (t[1]) vs. first orthogonal component (to[1]) of established OPLS-DA model based on the adrenal CS vs. CD dataset. The model parameters were as follows: *R* ^2^ *X* = 0.788, *R* ^2^ *Y* = 0.899, and *Q* ^2^ *Y* = 0.807. **(B)** Permutation test of 200 permutations of the OPLS-DA model. OPLS-DA, orthogonal partial least squares discriminant analysis.

To further study the underlying differences between the adrenal CS and CD groups, OPLS-DA was applied. An OPLS-DA model was established with one predictive and three orthogonal components (*R* ^2^ *X* = 0.788, *R* ^2^ *Y* = 0.899, and *Q* ^2^ *Y* = 0.807). The adrenal CS and CD groups exhibited a clear distinction, as shown in **Figure 3A**. The *Q* ^2^ *Y* value indicated the good predictability and good quality of the model. The results of statistical validation of the OPLS‐DA model by permutation analysis using 200 different model permutations showed that the built model is valid (**Figure 3B**).

**Figure 4:**
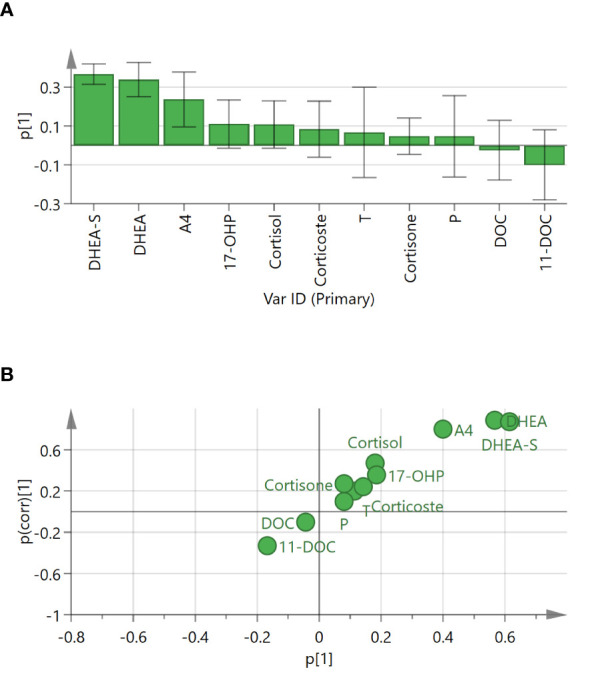
Loading plot and S-plot for identifying putative steroid biomarkers between adrenal Cushing syndrome (adrenal CS) and Cushing disease (CD). **(A)** P1 loading plot of the OPLS-DA model. p[1] provided confidence intervals of each loading value. **(B)** S-plot of OPLS-DA for adrenal CS and CD. The p[1]-axis describes the magnitude of each variable. The p(corr)[1]-axis represents the reliability of each variable. Ideal steroid biomarkers have high magnitude and high reliability. T, testosterone; DHEA, dehydroepiandrosterone; P, progesterone; 17-OHP, 17-OH-progesterone; A4, androstenedione; DOC, 11-deoxycorticosterone; 11-DOC, 11-deoxycortisol; DHEA-S, dehydroepiandrosterone-sulfate.

As shown in the P1 loading plot, DHEA-S, DHEA, and androstenedione were positively correlated with CD (**Figure 4A**). An S-plot (**Figure 4B**) and VIP were employed to reflect the importance of variables. Steroid biomarkers were selected based on the criterion VIP score > 1 and validated using *t*-tests. DHEA-S, DHEA, and androstenedione were identified as potential markers enabling differentiation between adrenal CS and CD (**Table 3**). DHEA-S and DHEA provided the best discrimination, followed by androstenedione.

**Table 3:** Summary of steroid biomarkers derived from the OPLS-DA model.

Biomarkers	VIP	AUC	p-value
DHEA-S ratio	2.03837	0.979	4.12062e^–8^
DHEA ratio	1.88414	1	5.79024e^–9^
A4 ratio	1.31677	0.944	9.51022e^–6^
17-OHP ratio	0.613463	0.667	0.126915
Cortisol ratio	0.598739	0.805	0.0150976
11-DOC ratio	0.558519	0.667	0.0685968
Corticosterone ratio	0.464982	0.621	0.141944
T ratio	0.373418	0.631	0.472274
Cortisone ratio	0.264856	0.595	0.141944
P ratio	0.262561	0.585	0.646301
DOC ratio	0.140212	0.505	0.703713

OPLS-DA, orthogonal partial least squares discriminant analysis; VIP, variable importance in the projection; AUC, area under the curve; T, testosterone; DHEA, dehydroepiandrosterone; P, progesterone; 17-OHP, 17-OH-progesterone, A4, androstenedione; DOC, 11-deoxycorticosterone; 11-DOC, 11-deoxycortisol; DHEA-S, dehydroepiandrosterone-sulphate.

A p-value < 0.05 was considered significant.

### 3.3 Sensitivity and specificity of the diagnosis of adrenal Cushing syndrome

A receiver operating characteristic curve analysis was used to assess the diagnostic power of plasma ACTH, serum DHEA-S, DHEA, A4, and its ratios in differentiating adrenal CS from CD (**Table 4**). The optimal cutoff value for ACTH from our patients was 12.6 pg/mL, with a sensitivity of 92.31% and a specificity of 100%. After excluding the patient with spuriously elevated ACTH, the range for ACTH was 5–12.6 pg/mL. The optimal cutoff value for ACTH remained at 12.6 pg/mL, with a sensitivity of 100% and a specificity of 100%. The DHEA-S ratio had a sensitivity of 92.31% and a specificity of 93.33%, with a cutoff value of 0.3971 and an area under the curve (AUC) of 0.979. The DHEA ratio had a sensitivity of 100% and a specificity of 100%, with a cutoff value of 0.1798 and an AUC of 1. The androstenedione ratio had a sensitivity of 92.31% and a specificity of 93.33%, with a cutoff value of 0.3752 and an AUC of 0.944. Their exact serum steroid concentrations showed similar diagnostic performances. A ROC analysis demonstrated that a DHEA-S ratio < 0.3971 (DHEA ratio < 0.1798) was sensitive and specific for the diagnosis of adrenal CS.

**Table 4:** Accuracy of steroid biomarkers for diagnosing adrenal Cushing syndrome.

	AUC (95% CI)	Sensitivity (%)	Specificity (%)	Youden index	Accuracy (%)
**DHEA-S ratio** **<** **0.3971**	0.979 (0.841–1.000)	92.31 (64.0–99.8)	93.33 (68.1–99.8)	0.8564	92.86
**DHEA-S ≤ 800,129.2737** **pg/mL**	0.974 (0.832–1.000)	92.31 (64.0–99.8)	93.33 (68.1–99.8)	0.8564	92.86
**DHEA ratio** **<** **0.1798**	1 (0.877–1.000)	100 (75.3–100.0)	100 (78.2–100.0)	1	100
**DHEA** **<** **907.4740** **pg/mL**	1 (0.877–1.000)	100 (75.3–100.0)	100 (78.2–100.0)	1	100
**A4 ratio** **<** **0.3752**	0.944 (0.786–0.995)	92.31 (64.0–99.8)	93.33 (68.1–99.8)	0.8564	92.86
**A4** **≤** **913.6404** **pg/mL**	0.918 (0.751–0.988)	92.31 (64.0–99.8)	86.67 (59.5–98.3)	0.7897	89.29
**ACTH** **≤** **12.6** **pg/mL**	0.938 (0.779–0.994)	92.31 (64.0–99.8)	100 (78.2–100.0)	0.9231	96.43

AUC, area under the curve; CI, confidence interval; ACTH, adrenocorticotropic hormone; DHEA-S, dehydroepiandrosterone-sulphate; DHEA, dehydroepiandrosterone; A4, androstenedione.

## 4 Discussion

The result of the PCA indicated that serum steroid profiles of adrenal CS patients were differentiated from that of CD patients. OPLS-DA is a multivariate statistical method, which was first proposed by Johan Trygg et al. in 2002, that identifies differences between two groups. It concentrates categorical information in one principal component while other data variations that have nothing to do with categorical variables or orthogonal variables are removed so that the model is easy and simple to explain. OPLS-DA showed obvious group discrimination and selected several steroids to discriminate adrenal CS from CD. DHEA-S, DHEA, and androstenedione were shown to be related to group separation, which is consistent with previous studies. Previous studies have found that serum DHEA-S and DHEA levels varied among different CS subtypes. Serum DHEA-S levels in Cushing syndrome patients due to adrenal adenoma were lower than that of patients with non-functional adrenal adenoma (10–12), whereas DHEA-S levels in CD patients were significantly higher than in the reference group (7, 13, 14). Significantly higher serum DHEA-S levels were confirmed in Cushing disease than in adrenal Cushing syndrome (7). Reduced DHEA-S levels were also described in patients with subclinical Cushing syndrome due to adrenal incidentalomas (11, 15–19). Steroid profiling by LC-MS revealed lower levels of DHEA and androstenedione in patients with subclinical CS than in patients with non-secreting adenomas and lower levels of DHEA in patients with non-secreting adenomas than in the control group in the basal condition (20). A retrospective study by Yener et al. suggested that a low DHEA-S value could be advantageous for distinguishing patients with subclinical CS. They retrospectively collected data from 249 subjects with adrenal incidentalomas, and 15.2% of participants met the criteria for subclinical CS after excluding overt CS and other adrenal gland diseases (16). DHEA-S levels were significantly reduced in subclinical CS patients compared with patients with non-functional adrenal adenomas. They reported that a DHEA-S value of 40.0 µg/dL was the best cutoff value for detecting subclinical CS (sensitivity = 68%; specificity = 75%; positive predictive value = 43%; negative predictive value = 90%; AUC = 0.788, and *p* < 0.001). Logistic regression showed DHEA-S (< 40.0 µg/dL) as the strongest predictor of the likelihood of having subclinical CS, with an odds ratio of 9.41.

Significantly reduced expression of dehydroepiandrosterone sulfotransferase in surgically removed attached non-neoplastic adrenal tissues in patients with subclinical CS and adrenal CS was described (11, 21), which was considered to represent the degree of suppression of the hypothalamus–pituitary–adrenal (HPA) axis. In patients with adrenal incidentalomas, a negative rank correlation between post-LDDST cortisol concentrations and serum DHEA-S was found, suggesting DHEA-S as a potential index in assessing subtle cortisol autonomy (22). It is also reasonable to find a lower level of DHEA-S in adrenal Cushing syndrome than in subclinical Cushing syndrome (23). A strong positive correlation between levels of DHEA-S and levels of ACTH and cortisol was detected in CD patients (24), supporting DHEA-S as a possible marker for the diagnosis of CD. Consistent with a previous study (7), CS patients had higher plasma levels of 11-deoxycorticosterone and 11-deoxycortisol than normal, whereas there were no differences for those steroids between patients with adrenal CS and CD. There were no differences in serum levels of progesterone, 17-OH-progesterone, and cortisone between the two groups, which is also consistent with previous study results (7). In contrast to a previous study (25), cortisol was shown to be significantly higher in CD than in adrenal CS in our study; however, the previous study found that the sum of cortisol metabolites and tetrahydrocortisol were significantly higher in CD than in adrenal CS, although this might only reflect a baseline difference of 24 h urinary free cortisol (25). Corticosterone tended to be higher in CD patients in a previous study (7), whereas in our study there was a significant difference. Of the 11 steroids analyzed, only DHEA-S, DHEA, and androstenedione showed a distinction between adrenal CS and CD after OPLS-DA.

Our results showed that the cutoff value of the DHEA-S ratio (DHEA-S concentration divided by the upper limit of the relevant reference range) for discriminating adrenal CS and CD was 0.40 and that the cutoff value for the DHEA ratio (DHEA concentration divided by the upper limit of the relevant reference range) was 0.18, with relatively high sensitivity and specificity. The result provided great insight into discrimination between adrenal CS and CD in respect of steroid profiles and the role it may play in clinical practice. One patient in our sample with falsely increased ACTH levels ultimately had a diagnosis of adrenal CS. She had repeated elevated plasma ACTH measurements of over 100 pg/mL (reference range: 0–46 pg/mL) and, combining results of other diagnostic tests, was considered to have ACTH-dependent Cushing syndrome. But after pituitary surgery her symptoms were not relieved. Her plasma ACTH level and 24-h urinary free cortisol were still high. Adrenal Cushing syndrome was her final diagnosis and was confirmed after unilateral adrenalectomy and pathological examination. Elevated ACTH levels were confirmed to be caused by heterophile antibodies. The DHEA and DHEA-S ratios were 0.0958 and 0.1553, respectively, below the diagnostic cutoff values in the current study. A recent study assessed the utility of DHEA-S to differentiate adrenal CS from CD, in which they only included patients with DHEA-S levels within the reference interval, and reported that a DHEA-S percentile rank of 19.5% had a sensitivity of 80.8% and specificity of 81.5% for the differential diagnoses of CD and adrenal CS (26). Hána et al. generated a prediction model of ACTH-dependent compared with ACTH-independent CS, finding that DHEA could best differentiate ACTH-dependent CS from ACTH-independent CS (27).

Although plasma ACTH level is used first to discriminate between adrenal CS and CD, the overlap in values between adrenal CS and CD (4) and falsely elevated ACTH levels (5) could mislead the direction of differential diagnoses. Falsely elevated ACTH levels lead to the consideration of ACTH-dependent CS, also leading to screening for pituitary adenoma or ectopic ACTH-secreting adenoma. In addition, incidental pituitary tumors were found to be disclosed on MRI in 10% of the general population (2) and patients with adrenal CS could harbor a pituitary adenoma simultaneously (4), making it more obscure to find the real etiology in CS patients. It is important to realize that healthy volunteers could have increased inferior petrosal sinus-to-peripheral ratios, as they can have ACTH responses to CRH (5), that is, a positive inferior petrosal sinus sampling (IPSS) result is not sufficient to suggest that a pituitary adenoma is the etiology. As a seemingly “positive” IPSS may present in patients without a pituitary source of ACTH (5), an elevated ACTH result may converge to cause misdiagnosis or result in unnecessary pituitary surgery. With more cases with the ACTH assay problem being reported, Greene believed these cases to represent a group of cases that are unrecognized or unreported (5). The possibility of spurious elevated ACTH results should be considered in clinical practice and adjuvant diagnostic information could be helpful.

The results revealed that DHEA-S and DHEA were the most prominent steroids for differentiating adrenal CS from CD. Owing to the relationship between DHEA-S and ACTH, if DHEA-S and DHEA do not match the result of spuriously elevated ACTH, ACTH test accuracy and ACTH-independent CS should be highly suspected. A further differential diagnosis of ACTH-dependent CS may be unnecessary. We thought that DHEA-S and DHEA tests could be useful to help rule out the consideration of ACTH-dependent CS when serum ACTH and DHEA-S (DHEA) mismatch, especially when ACTH is spuriously elevated and/or both a pituitary lesion and adrenal adenoma are present in one patient. Owing to the small sample size of our study, more accurate cutoff values of DHEA-S and DHEA for differentiating adrenal CS from CD should be confirmed in prospective large sample size studies and various ethnicities. We did not include ectopic ACTH syndrome cases; the diagnostic utility of DHEA-S and DHEA in differentiating adrenal CS from ectopic ACTH syndrome should be validated in future studies. In addition, we did not include primary bilateral macronodular adrenal hyperplasia (PBMAH) cases in this study. It is well established that ACTH can be produced by some adrenocortical cells, influencing steroid production directly and indirectly (28, 29). The level of DHEA-S might be influenced consequently, and whether the levels of DHEA-S and DHEA in PBMAH are in accord with that of hypercortisolism due to adrenocortical adenomas should be verified.

DHEA-S has a long half-life and is relatively stable throughout the day (17), making its measurement a useful additional test to confirm the source of etiology in cases with falsely elevated ACTH results. In conclusion, the study suggested that DHEA-S, DHEA, and androstenedione were the steroids differing between adrenal CS and CD and their measurement might serve as an additional diagnostic test, especially when the ACTH test result is high.

## Data availability statement

The raw data supporting the conclusions of this article will be made available by the authors, without undue reservation.

## Ethics statement

The studies involving human participants were reviewed and approved by Institutional Review Board of Tianjin Medical University General Hospital. The ethics committee waived the requirement for written informed consent for participation.

## Author contributions

CG, LD, and XZ designed research, analyzed the data, and wrote the manuscript. MY, ST, WL, and YY researched the data. QH and ML designed the study, and revised and edited the manuscript paper. All authors contributed to the article and approved the submitted version.
